# Inflammatory responses and intestinal injury development during acute *Trypanosoma cruzi* infection are associated with the parasite load

**DOI:** 10.1186/s13071-015-0811-8

**Published:** 2015-04-03

**Authors:** Bruna Perez Vazquez, Thaís Perez Vazquez, Camila Botelho Miguel, Wellington Francisco Rodrigues, Maria Tays Mendes, Carlo José Freire de Oliveira, Javier Emílio Lazo Chica

**Affiliations:** Curso de Pós-graduação em Ciências da Saúde, Universidade Federal do Triângulo Mineiro, Uberaba, 38025-180 Minas Gerais Brazil; Disciplina de Biologia Celular/Instituto de Ciências Biológicas e Naturais, Universidade Federal do Triângulo Mineiro, Uberaba, 38061-500 Minas Gerais Brazil; Curso de Pós-Graduação em Medicina Tropical e Infectologia, Universidade Federal do Triângulo Mineiro, Uberaba, 38015-050 Minas Gerais, Brasil

**Keywords:** *T. Cruzi*, Chagas disease, Parasite load, Intestinal injury, Inflammation

## Abstract

**Background:**

Chagas disease is caused by the protozoan *Trypanosoma cruzi* and is characterized by cardiac, gastrointestinal, and nervous system disorders. Although much about the pathophysiological process of Chagas disease is already known, the influence of the parasite burden on the inflammatory process and disease progression remains uncertain.

**Methods:**

We used an acute experimental disease model to evaluate the effect of *T. cruzi* on intestinal lesions and assessed correlations between parasite load and inflammation and intestinal injury at 7 and 14 days post-infection. Low (3 × 10^2^), medium (3 × 10^3^), and high (3 × 10^4^) parasite loads were generated by infecting C57BL/6 mice with “Y”-strain trypomastigotes. Statistical analysis was performed using analysis of variance with Tukey’s multiple comparison post-test, Kruskal–Wallis test with Dunn’s multiple comparison, χ2 test and Spearman correlation.

**Results:**

High parasite load-bearing mice more rapidly and strongly developed parasitemia. Increased colon width, inflammatory infiltration, myositis, periganglionitis, ganglionitis, pro-inflammatory cytokines (e.g., TNF-α, INF-γ, IL-2, IL-17, IL-6), and intestinal amastigote nests were more pronounced in high parasite load-bearing animals. These results were remarkable because a positive correlation was observed between parasite load, inflammatory infiltrate, amastigote nests, and investigated cytokines.

**Conclusions:**

These experimental data support the idea that the parasite load considerably influences the *T. cruzi*-induced intestinal inflammatory response and contributes to the development of the digestive form of the disease.

## Background

Chagas disease (CD) is an anthropozoonosis caused by the protozoan *Trypanosoma (T.) cruzi* [1,2]. CD is endemic in Central and South America, where an estimated 7–8 million individuals are affected and an additional 25 million individuals are at risk of contracting the disease [2,3]. CD occurs via a multifactorial process, given the diversity of factors related to the host–parasite relationship [4]. Clinically, CD usually develops from an acute to a possibly debilitating chronic phase. Although the majority of patients remain clinically asymptomatic for many years (e.g., 20–30 years), the chronic phase of this disease can include cardiac, digestive, cardiac/digestive, or nervous system manifestations [5-8].

In the digestive form of the disease, megaesophagus and megacolon have been described as the primary manifestations resulting from gastrointestinal tract lesions [9]. The rectum and sigmoid colon are the most compromised segments [10] and exhibit striking luminal enlargement and muscular hypertrophy. Inflammatory lesions in the enteric nervous system are associated with substantial reductions in the numbers of neurons. This neuronal loss is thought to underlie the clinical findings observed with mega syndromes [11].

The digestive manifestations of CD are quite diverse, and this diversity is related to the host immune response, environmental and genetic factors, and the parasite itself. More specifically, it has been suggested that the characteristics of the immune response during the acute phase might be associated with the intensity and severity of the clinical signs observed during the chronic disease phase. To give an example, intestinal myenteric denervation caused by interferon-dependent nitric oxide production during the acute disease phase has been shown to be critical for the development of the digestive form of the disease during the chronic phase [12]. Moreover, altitude, ethnicity, drug use, and diet have also been suggested to influence the size and length of the rectal and sigmoid colon segments of the patient’s intestine [13]. Experimental morphometric studies that evaluated the effects of 2 different parasite inocula in Swiss mice demonstrated that reductions in the numbers of myenteric nerves occurred mainly between 30 and 75 days of infection; this finding was linked to the parasite inoculum and infection duration [14]. More recently, our group experimentally demonstrated that cardiac an renal manifestations of CD were associated with the parasite burden and the inflammation resulting from infection [15,16], and these findings suggest that changes in other organs such as the intestine might be also affected by the same parameters. Lately it has been rated the strains of *T. cruzi* according to their genetic characteristics, and our work reinforces that besides the pathogenic characteristics attributed to each Discrete Typing Unit (DTU) of T. cruzi [17-19], it is also important to consider the concentration of the inoculum in the induction of these intestinal disease processes.

To date, published studies in which the digestive manifestations of CD were evaluated have only assessed the effect of a single parasite load; in studies that evaluated the effects of 2 parasitic loads, only reductions in the numbers of intestinal myenteric nerves were assessed. Because the digestive manifestations of CD correlate with both the host immune response and the parasite, we sought to evaluate whether different parasite loads would differentially influence the parasite burden, inflammatory process, and the progression of intestinal symptoms. Moreover, we assessed the correlations between the parasite load, inflammation, and intestinal injury using an acute experimental disease model.

## Methods

### Animals

Male C57BL/6 mice (6–8 weeks of age) weighing 20–30 g were housed in temperature-controlled rooms (22-25 °C) with access to water and food (Nuvilab-CR1, NUVITAL - Nutrients Veterinary Products Ltda – Curitiba, PR, Brazil) ad libitum in the animal facilities of the Laboratory of Cell Biology, Institute of Biological and Natural Sciences, Federal University of Triângulo Mineiro (UFTM), Uberaba, Minas Gerais, Brazil. The protocol for all experiments involving mice was evaluated and approved by the UFTM Institutional Animal Care and Use Committee (protocol number 145/2010). None of the mice were used in more than 1 experimental group. The mice used in this study were divided into the following groups: uninfected or infected with 3 × 10^2^ (low), 3 × 10^3^ (medium) or 3 × 10^4^ (high) trypomastigotes.

### Parasite strain and mouse infection

The “Y” strain of *T. cruzi* was used in these experiments. C57BL/6 mice (10 animals per group) were infected subcutaneously with a blood-derived trypomastigote strain (MHOM/BR/00Y; *T. cruzi* II) according to the above-listed group descriptions [20,21]; this strain was kindly provided by the University of São Paulo (Brazil) and maintained in the Department of Cell Biology at UFTM.

### Parasitemia and survival

Parasitemia was quantified in the infected mice according to Brener’s technique [22]. Briefly, parasites were counted in 50 microscopic fields of a wet preparation containing 5 μl of blood collected from tail snips. Microscopic blood parasite examinations were performed daily until the day 14 of infection, and the results were expressed as parasites/mL. In other experiments, mice were infected with 3 × 10^2^, 3 × 10^3^, or 3 × 10^4^ trypomastigotes, and the deaths were recorded daily.

### Histological and immunohistochemical analysis

Before necropsy, mice were sacrificed in a CO_2_ chamber on days 7 and 14 after *T. cruzi* infection. For histological processing, the distal segment of the large intestine was removed, planed, and fixed on filter paper in formaldehyde for 24 hours, followed by storage in 70% alcohol until further use. The intestines were subjected to processing via dehydration, inclusion, and diaphanization followed by microtomy. The blocks were cut on a rotary microtome to obtain 5-μm sections. The sections were placed on slides (10 slides per intestine), and the procedure was repeated until all slides contained 4 sections each (at 25-μm intervals). This procedure was performed 10 times without discarding any slices. After drying at 60 °C, the sections were stained with hematoxylin/eosin (HE). Images were obtained using a light microscope plus camera (Nikon – Eclipse 50i) in a magnification of 20x. The evaluations were performed using ImageJ software (http://rsb.info.nih.gov/ij/). The thickness and width of the intestines were calculated to evaluate the structural morphology. In addition, we measured the areas of myositis, ganglionitis, and periganglionitis relative to the area of the muscle layer of the colon.

Furthermore, sections were mounted on glass slides and used for immunohistochemical analyses. The slides were pre-treated with 3-aminopropyltriethoxy-silane (Sigma-Aldrich Corporation, St. Louis, MO, USA). The sections in glass slides were immersed in xylene for 10 min to eliminate the paraffin, dehydrated in absolute alcohol, and re-hydrated with Tris-buffered saline (TBS). The sections were rinsed in TBS and immersed in a 3% hydrogen peroxide–methanol solution for 30 min to block endogenous peroxidase activity, followed by a 30-min incubation at 90 °C in the same solution for antigen retrieval. *T. cruzi* antigen immunolabeling was performed with an antibody raised in rabbits (1:250 dilution); the slides were incubated with this antibody for 2 h at 37 °C and subsequently rinsed thrice with TBS for 3 min per wash. Next, the slides were incubated with peroxidase-conjugated protein A (1:100) for 1 h at room temperature. The slides were washed again and treated with 3,3-diaminobenzidine tetrahydrochloride (DAB Chromogen Kit; Biocare Medical, Concord, CA, USA) for imaging. The slides were subsequently counterstained with Mayer’s hematoxylin and mounted. The primary antiserum was omitted from the control sample to account for non-specific staining.

### Tissue extract preparation for cytokine measurements

The TNF-α, IFN-γ, IL-2, IL-6, IL-17, and IL-10 concentrations in the intestinal tissues were analyzed on days 7 and 14 after *T. cruzi* infection. The intestinal tissues were first weighed using a precision balance (GEHAKA, model BG 4400, SP - Brazil) and then immersed in equal volumes of phosphate-buffered saline (PBS; 500 μL per tissue) containing protease inhibitor (complete Protease Inhibitor Cocktail Tablets; Roche Applied Sciences, Indianapolis, IN, USA). The protease inhibitor solution was prepared by adding 1 tablet to 50 mL of PBS according to the manufacturer’s instructions. Extracts were obtained by homogenizing tissues (Ultra Turrax®, Wilmington, NC, USA) in protease inhibitor buffer with an electrical tissue homogenizer, followed by centrifugation at 300 × g for 15 min, after which the supernatants were collected and stored at −70° until use. The cytokine bead array technique (CBA – BD Biosciences) was used for cytokine detection and quantification, and the samples were analyzed via flow cytometry (FACSCalibur^TM^, BD Biosciences, San Jose, CA, USA) and CellQuest software (BD Biosciences) according to the manufacturer’s instructions. The cytokine concentrations were normalized according to the weight of each tissue, and the results were expressed as pg/mg of tissue.

### Statistical analysis

The statistical analysis was performed using the Prism software program (GraphPad Inc., San Diego, CA, USA). Normality (Kolmogorov–Smirnov test) and homogeneous variance tests (Bartlett’s test) were applied to all variables. Parametric tests (analysis of variance with Tukey’s multiple comparison post-test) were used for cases with normal distributions and homogeneous variances, and the results were expressed as means ± standard errors of the mean (SEM). Non-parametric tests (the Kruskal–Wallis test with Dunn’s multiple comparison) were used for cases with non-Gaussian distributions, and the results were expressed as median, maximum, and minimum values. The chi-squared (χ^2^) test was used to compare the frequencies of amastigote nest positivity among the different groups (expressed as %). The Spearman non-parametric rank test was used to correlate the data. Differences with p-values <0.05 (5%) were considered significant.

## Results

### Effects of the parasite load on parasitemia and the survival of *T. Cruzi*-infected mice

**Figure 1:**
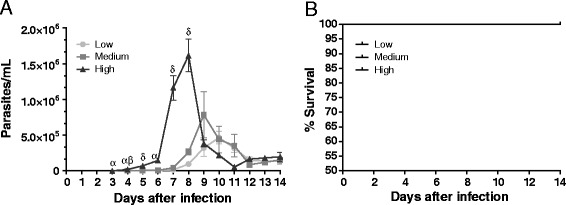
***Effects of different T. cruzi inoculum concentrations on parasitemia and survival*** **.** C57BL/6 mice were challenged with 3 × 10^2^ (low dose), 3 × 10^3^ (medium dose), or 3 × 10^4^ (high dose) blood trypomastigotes (strain “Y”). Parasitemia **(A)** was determined by counting the number of parasites in 5 μL of blood collected from tail snips at the indicated time points. Each point represents the mean ± standard error of the mean of the individual values from 10 mice. In the survival curve **(B)**, 10 animals were individually monitored for a 14-day infection period. α indicates a significant (p < 0.05) difference when comparing the mice infected with high doses to those infected with medium and low doses, β indicates a significant (p < 0.05) difference when comparing the mice from the medium-dose group to those from the low-dose group, and δ indicates a significant (p < 0.05) difference when comparing the mice infected with high doses to those infected with low doses. Nonparametric tests were used for analysis of graphics listed here.

The parasitic blood levels and survival of *T. cruzi*-infected animals are important parameters with which to determine the points of the study and facilitate a consequent understanding of the host–parasite relationship. Therefore, to investigate the effects of the parasite load on parasitemia and survival, mice were inoculated with 3 × 10^2^ (low), 3 × 10^3^ (medium) and 3 × 10^4^ (high) trypomastigote forms prior to measuring these important parameters. In the group inoculated with 3 × 10^4^ parasites, the parasitemia (Figure 1A) had already begun on day 3 after infection and this parasitemia exhibited a significant increase (p < 0.05) when compared with that in the other groups on days 3, 4, and 6 after infection. On days 7 and 9 after infection, the high-infection group exhibited a significant difference in parasitemia only when compared with the low-infection group (p < 0.05). On day 7 after infection, the low and medium groups began to exhibit an exponential increase in the number of parasites in the blood and on day 14 of infection, the 3 groups presented with the same levels of parasites in the blood (Figure 1A). The animals infected with low, medium, and high trypomastigote loads survived throughout the 14-day experimental period (Figure 1B).

### Morphometric analysis of the intestinal tissues from *T. Cruzi*-infected mice

**Figure 2:**
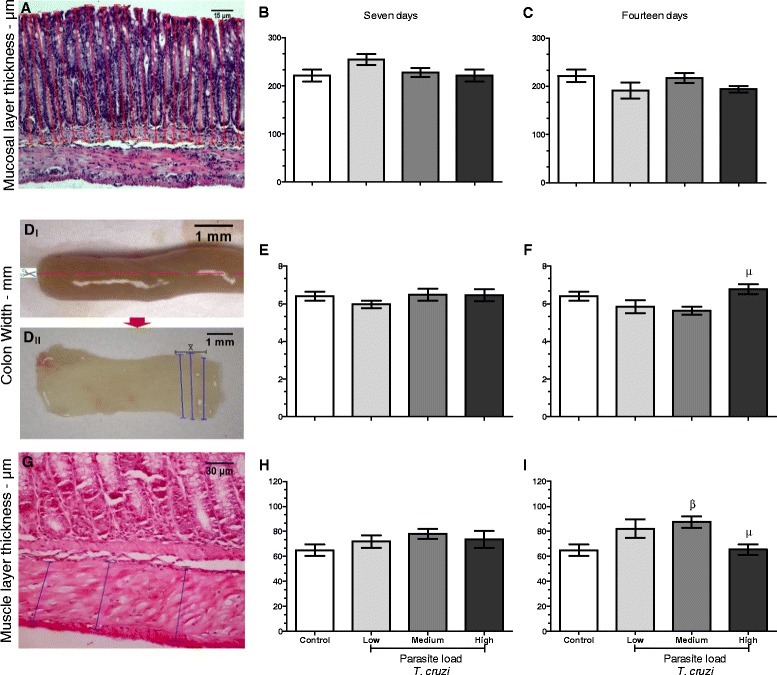
**Parameters of the intestinal epithelium width and thickness in mice with different** ***T. cruzi*** **concentrations.** At 7 and 14 days after infection with 3 × 10^2^ (low dose), 3 × 10^3^ (medium dose) or 3 × 10^4^ (high dose) blood trypomastigotes, mice were sacrificed in a CO_2_ chamber, followed by the removal of the distal segment of the large intestine. The intestines were washed with phosphate-buffered saline (1x), planed on filter paper, and fixed in formaldehyde for 24 hours, followed by processing as described in the Materials and Methods. After staining with hematoxylin–eosin, images were captured and analyzed to determine the mucosal layer thickness **(A–C)**; the red line determines the measurement region. Colon width was evaluated (D–F), in D_I_, the dotted line shows sectional area for planed the intestinal tubular structure, in D_II_, planed fragment, the blue line determines the location of measurement where was extracted from the mean (Ẋ). Muscle layer thickness too was determinate **(G–I)**, the blue line determines the measurement region. μ indicates a significant (p < 0.05) difference when comparing the mice infected with high parasite doses to those infected with a medium dose, and β indicates a significant (p < 0.05) difference when comparing the mice infected with a medium dose to uninfected mice. Parametric tests were used for analysis of graphics listed here.

Changes in the intestinal architectural parameters comprise a characteristic often associated with the digestive form of CD in both the acute and chronic phases. To investigate whether the parasite load might influence changes in these parameters, we measured the mucosal layer thickness, colon width, and muscle layer thickness. Regarding the mucosal layer thickness (Figure 2A–C), we did not detect significant changes during the evaluation period. Regarding the colon width (Figure 2D–F), we observed that at day 7 after infection, no significant differences were found but that after blood parasite multiplication and the consequent decrease of parasites in the blood (day 14 after infection) a significant increase (p < 0.05) in this parameter was observed in mice infected with a high inoculum dose. After analyzing the gut muscle layer thickness (Figure 2G–I), we observed a significant increase in this parameter in mice that had been infected with a medium inoculum dose (p < 0.05); however, we found that mice infected with a high inoculum dose exhibited a decrease in the muscle layer thickness relative to that of the medium group. The values of the measurements taken for this parameter can be best viewed in Table 1.

**Table 1:** **Histometric data**

	**Control**	***T.cruzi*** **-infection**							
		**7th day after**			**P-value**	**14** ^**th**^ **day after**			**P-value**
		**Low**	**Medium**	**High**		**Low**	**Medium**	**High**	
McLT - μm	221.8	255.10	228.30	222.00	0.22	191.10	217.60	194.30	0.16
Std. Error	12.80	11.83	9.44	12.39		16.47	10.32	6.93	
CE (%)	5.77	4.64	4.13	5.58		8.61	4.74	3.57	
CL - mm	6.40	5.97	6.48	6.46	0.54	5.85	5.63	6.78^μ^	0.02
Std. Error	0.23	0.21	0.32	0.31		0.35	0.21	0.26	
CE (%)	3.59	3.52	4.94	4.80		5.98	3.73	3.83	
MsLT - μm	64.88	71.78	78.13	73.56	0.31	82.04	87.49^β^	65.42^μ^	< 0.01
Std. Error	4.63	4.84	3.99	6.87		7.50	4.64	4.25	
CE (%)	7.14	6.74	5.11	9.34		9.14	5.30	6.50	

McLT = Mucosal Layer Thickness, CL = Colon Width, MsLT = Muscle Layer Thickness, Std. Error = Standard Error, CE = Coefficient of Error, μ = p < 0.05 high vs medium dose, β = p < 0.05 medium dose vs uninfected.

### Effects of *T cruzi* inoculum concentration on the appearance of myositis, ganglionitis, and periganglionitis in the intestinal tissues of *T. Cruzi*-infected mice

**Figure 3:**
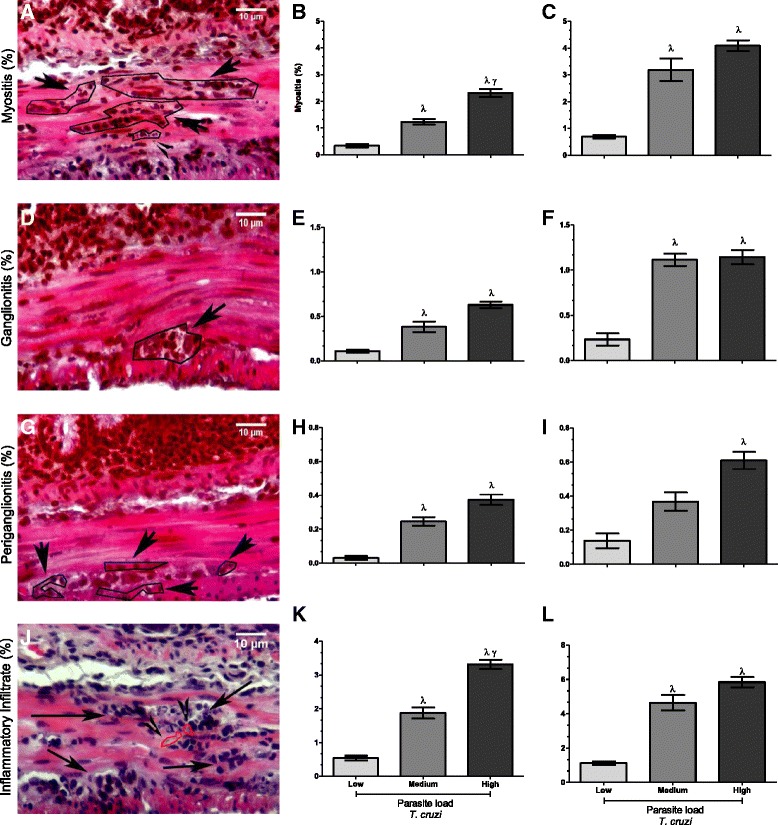
**Analysis of the inflammatory infiltrates in the intestinal tissues.** C57BL/6 mice were challenged with low, medium, and high trypomastigotes doses and at 7 and 14 days post-infection, the inflammatory infiltrates were evaluated. The percentages of inflammatory distribution in the intestinal muscle layer **(A–C)**, ganglia **(D–F)**, near the ganglia **(G–I)**, and the entire intestinal tissue were determined **(J–L)**. Arrows indicate sites of inflammation and the arrowheads indicate the presence of forms amastigotes of *T. cruzi*. λ indicates a significant (p < 0.05) difference between the mice infected with a low dose and γ indicates a significant (p < 0.05) difference between the mice infected with a medium dose. Parametric tests were used for analysis of graphics E and H, and nonparametric tests for the graphics **A**, **B**, **D**, **G**, **J** and **K**.

As *T. cruzi* is known for its ability to alter the intestinal architecture, we conducted an assay to assess whether the parasite load would differently induce the inflammatory processes in the muscle layer, ganglion, and periganglionic areas of the intestinal architecture. We observed in a dose-dependent manner that by day 7 after infection, the group that had received a high inoculum dose exhibited increased myositis relative to the medium and low groups. A representative image of myositis in an infected group infected is shown in Figure 3A. In mice from the medium group, myositis was also significantly increased relative to that in mice from the low group (p < 0.05; Figure 3B). At day 14 after infection, we also observed statistically significant increases in myositis in the medium and high groups relative to the low group (Figure 3C). Similarly, when we evaluated infiltration in the ganglions (Figure 3D–F), we observed a larger area of ganglionitis in tissues from the medium and high groups, and this difference was statistically significant relative to the low group. When we evaluated periganglion infiltration (Figure 3G–I), which increased numbers of cells around the ganglion can be visualized in Figure 3G, increased levels of inflammatory infiltrate were found in the samples from the medium and high groups by day 7 after infection (Figure 3H), and this event was also evident in the high group at day 14 after infection (Figure 3I). When we evaluated inflammatory infiltration in the intestinal tissue, we observed a very similar parasite load-dependent induction of inflammation (Figure 3 J–L). The inflammatory infiltration in the large intestinal muscle layer can be seen in Figure 3J. As shown in the figure 3K, at day 7 after infection significant increases in infiltration could be observed in the high group relative to the other groups and in the medium group relative to the low group (p < 0.05). At 14 days after infection, the medium and high groups exhibited similar levels of inflammatory infiltrate, both of which were significantly higher than the level observed in the low group (Figure 3L).

### Influence of *T cruzi* inoculum concentration on amastigote nest formation in the intestinal tissues

**Figure 4:**
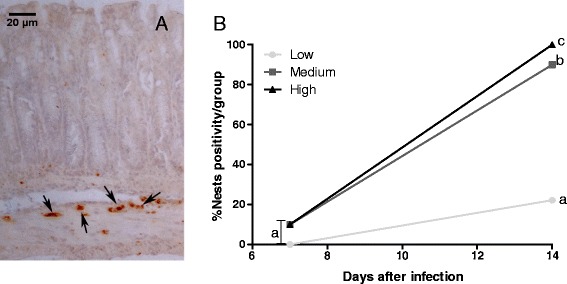
**Immunohistochemical analysis of** ***T. cruzi*** **in the intestinal tissues of different groups.** Amastigote nest positivity was evaluated according to the presence of intestinal tissue nests in 10 animals per group (low, medium, and high doses). Immunohistochemistry was performed with antibodies against *T. cruzi*, and the subsequent immune complexes were visualized with the substrate diaminobenzidine. Images were obtained using a 20× objective lens. Antigenic staining of amastigote nests was observed in the intestinal tissues, as indicated by arrows **(A)**. A comparison of nest positivity according to group (in percentages) at 7 and 14 days after infection **(B)**. The groups were compared on the different days and between the different days. The letters a, b and c indicate statistically significant differences between them (p < 0.05). Chi-squared test was used here.

The initiation of cell migration to the infection site might be caused by the presence of antigen. Therefore, after demonstrating that the intestinal inflammatory infiltrate was dependent on the parasite load, we evaluated the presence of amastigote nests in the intestinal tissues at 7 and 14 days after infection. Brownish markings in Figure 4A indicate positivity in detection by immunohistochemistry to the presence of amastigotes of *T. cruzi* in the intestinal tissue. The presence of amastigote nests in the intestinal tissues was observed by day 7 after infection in the mice inoculated with medium and high parasite loads (Figure 4B). At day 14 after infection, all groups of mice had developed amastigote nests in the intestinal tissues but the groups that had received the highest inoculum dose had significantly increased nest formation relative to the group that had received the lowest inoculum dose (Figure 4B).

### Influence of *T cruzi* inoculum concentration on inflammatory cytokine production in the intestinal tissues of mice acutely infected with *T. Cruzi*

**Figure 5:**
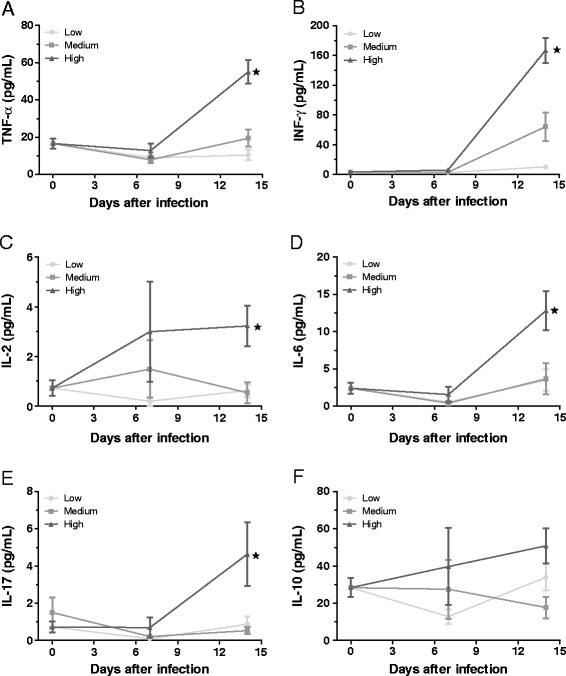
**Determination of cytokine concentrations in the intestinal tissues.** The TNF-α **(A)**, IFN-γ **(B)**, IL-2 **(C)**, IL-6 **(D)**, IL-17 **(E)**, and IL-10 **(F)** concentrations in intestinal tissue slices were determined on days 0 (without infection), 7, and 14 after *T. cruzi* infection in mice that received low, medium, and high parasite doses. The samples were obtained by homogenizing tissues in a protease inhibitor buffer, followed by centrifugation at 300 × g for 15 min. A cytokine bead array was used for cytokine detection and quantification, and the samples were analyzed by flow cytometry with the CellQuest software as described in the Materials and Methods. The groups were evaluated on separate days. * indicates a significant (p < 0.05) difference between the groups. Parametric tests were used for analysis to time 0 of all cytokines, to time 7 of IL-10, and to time 14 of IL-10, TNF- α, IL-6 and IL-2, and nonparametric tests for other ratings.

An excellent way to associate parasitism with an increase in the number and activity of inflammatory cells within infected tissues is to evaluate cytokine production at that site. To determine this association, we quantified the effect of the parasite load on the production of important inflammatory cytokines in tissues both before and at 7 and 14 days after infection. We conducted comparisons between the groups at different periods of infection (0, 7, and 14 days). After 7 days of infection, we observed an imbalance in the cytokine concentrations between the different groups; however, these differences were not significant (p > 0.05). In contrast, at day 14 after infection, increased levels of the pro-inflammatory cytokines TNF-γ, IFN-γ, IL-2, IL-6, and IL-17 (Figure 5A–E) were observed in mice that had received a high inoculum dose relative to mice that had received medium and low doses. In addition, there were no significant differences between the groups with respect to IL-10 production (Figure 5F).

### Effect of *T. Cruzi* strain "Y" infection on the correlation between inflammatory infiltration, amastigote nests, and proinflammatory cytokines in the intestinal tissues

**Figure 6:**
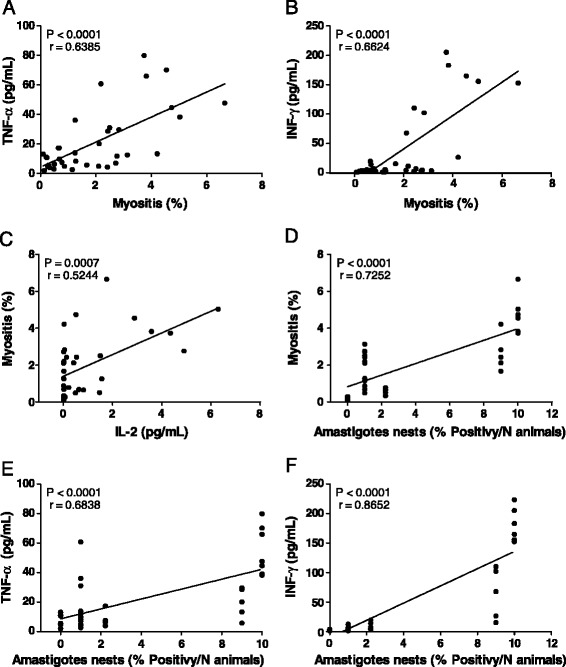
**Correlation between inflammatory infiltration, amastigote nests, and cytokines. (A)** The relationship between the TNF-α concentration and inflammatory myositis. **(B)** The relationship between the INF-γ concentration and myositis. **(C)** The relationship between myositis and the IL-2 concentration. **(D)** The relationship between myositis and amastigote nests. **(E)** The relationship between the TNF-α concentration and amastigote nests. **(F)** The relationship between the INF-γ concentration and amastigote nests. Data are representative of 2 independent experiments. Statistical analysis: Spearman correlation.

Finally, we analyzed the data to determine whether the results reported herein would correlate significantly. The correlations between TNF-α production and myositis (Figure 6A), INF-γ production and myositis (Figure 6B), IL-2 production and myositis (Figure 6C), myositis and amastigote nest formation (Figure 6D), TNF-α production and amastigote nest formation (Figure 6E), and INF-γ production and amastigote nest formation (Figure 6F) were all positive (Spearman correlation, p < 0.05).

## Discussion

This report has revealed that the gastrointestinal tract is damaged during the first days of an experimental acute *T. cruzi* infection and that this damage is more pronounced in mice that have been infected with high parasite loads. Thus, we demonstrated that mice acutely infected with high parasite loads exhibited a significant differences with respect to the parasitemia onset and peak and that increased colon width, intestinal inflammatory infiltration, myositis, periganglionitis, ganglionitis, pro-inflammatory cytokine production (TNF-α, INF-γ, IL-2, IL-6I, L-17) and amastigote nest formation was also observed mainly in animals infected with high parasitic loads. These results were so remarkable that positive correlations were found between the parasite load and inflammatory infiltration, amastigote nest formation, and the investigated cytokines.

First, we demonstrated that during a 14-day infection period, the logarithmic *T. cruzi* trypomastigote concentration affected the parasitemia onset, peak, and time of initial decline. The survival rate was not altered during this period. As previously demonstrated by other authors, the parasite concentration at the time of infection affects the development of parasitemia [15,23,24], and this phenomenon might be explained by the parasite cycle, a host immune response that favors binary division in animals that received the highest parasite load, or a stronger anti-parasitic response in animals that received the lowest parasite load [15]. With respect to survival, the absence of death can be explained by the short evaluation period (14 days), as previous studies using the same methodology and parasite strain reported that mice began to die at 18 days after infection and that this mortality was observed only in mice infected with the highest parasite load (3 × 10^4^ trypomastigote forms) [15].

Besides parasitism, CD is characterized by the presence of inflammation and lesions in many organs, including the heart, kidney, and gut. It has been well established that inflammation may occur in various tissues, regardless of the affected organ. Herein, we demonstrated that *T. cruzi* affected the colon width and muscle layer thickness but that these effects were only significant at 14 days after infection and in mice inoculated with high and medium parasite doses, respectively. Furthermore, the incidence of myositis, ganglionitis, periganglionitis, and inflammatory infiltration, taken together, increased from day 7 after infection, and these 4 parameters also increased in a parasitic load-dependent manner. It is important to emphasize that the initial inflammatory process did not vary in the studied tissues because the medium and high parasite loads induced strong inflammation in the intestinal tissues independently of the inflammatory foci. Based on these experimental data, we suggest that the inflammatory process that occurs after day 7 of infection remains insufficient to induce lesions in the intestinal tissues; however, this persistent inflammatory process affects the colon width and muscle layer thickness, as observed on day 14.

Empowering deleterious factors in the acute phase of the disease by increasing the number of parasites in the inoculum contributed significantly to reducing the thickness of the muscular layer, event observed when we compared the medium and high groups. Morphological changes in intestinal tissue associated with Chagas disease are already known and widely studied in the chronic phase of the disease [13,25,26]; since the morphometric changes are associated with denervation of the muscle layer, which facilitates the dilation of the tissue, thereby decreasing its thickness. Accordingly, we add new information to the literature since we have demonstrated that a reduction in thickness also happens in the acute phase due to an increase in the inoculum. In other words we reinforce that the intestinal damage are directly related to the inoculum and the time of infection [14].

Our results were quite similar to experimental findings already published in the literature; for example, a previous report demonstrated that at 30 days post-infection, mice infected with 10^4^ trypomastigote forms of the “Brasil” strain exhibited myositis and ganglionitis [27]. Moreover, dogs infected with different *T. cruzi* strains also exhibited inflammatory processes in the gut during both the acute and chronic phases [28]. Is related in chronic chagasic patients the presence of myositis and ganglionitis, but these events are more frequent and intense in organs with megacolon when compared to those without megacolon [29]. In the case described here, this evidence was very important, because the presence of inflammation (myositis and ganglionitis) was already observed on day 7 after infection and had intensified by day 14. In most studies involving *T. cruzi*, inflammatory processes in the gut have been poorly described, especially when day 7 of infection has been evaluated. We believe that this discrepancy is mainly due to the fact that most studies work with low numbers of inoculated trypomastigotes at the moment of infection. As you can see from our findings, mice infected with higher numbers of parasites developed more severe inflammation. Thus, it was possible to verify that the intestinal tissues from *T. cruzi*-infected mice developed light, medium, or very intense inflammatory processes depending on the initial parasite load.

Regarding cytokine production, our findings indicated that the gut immune responses in *T. cruzi*-infected mice did not follow a polarized Th1- or Th17-type pattern. In other words, the production of cytokines associated with both patterns had increased by day 14 in the animals that had received a higher parasitic load. The role of IL-17 in the immune response to *T. cruzi* has gained prominence in recent years, as independent research groups have shown that this cytokine is an important component of host defense during the acute disease phase [30-32]. Here, we demonstrated the same biologic event; however, this cytokine was only induced in animals that had received a high parasite load, a factor that was not evaluated in previous studies. Similar to IL-6, elevated IL-17 levels have been found in mice receiving high parasite loads, and this finding emphasizes the relationship between the production of these inflammatory cytokines and the development of CD [33]. The importance of IL-2, IFN-γ, and TNF-α has been known for nearly 2 decades [34-37], and both early and later studies have shown that these cytokines, if uncontrolled by regulatory T cells or anti-inflammatory cytokines such as IL-10, play crucial roles in both protection and disease development and progression [38-41].

Slight IL-10 production was also observed in animals receiving the highest parasite doses; however, there were no significant differences with respect to the evaluated days and concentrations. Our results were unexpected and differed from the findings of other investigators regarding IL-10 in organs other than the intestine [15]. We expected that the production of this cytokine would be increased even in animals that had received the lowest parasite loads, as the gut has a higher propensity for controlling pro-inflammatory immune responses by producing IL-10. It is known that during intracellular pathogen-mediated infections, increased pro-inflammatory cytokine production is accompanied by IL-10 production [37,42]. Furthermore, the production of this, anti-inflammatory cytokine was shown to increase in different affected organs, including the heart and kidney, and this production was found to be induced in animals infected with low and high parasitic loads [15,43-45].

It is important to highlight that both inflammation and the number of amastigotes in the intestinal tissues are associated with the time of infection and parasitemia; in another words, as depicted in Figure 4, the time of infection and parasite load also affected the presence of amastigote nests in the gut and, consequently, the presence of inflammation and lesions in the intestinal tissues. Therefore, the findings of this study revealed positive correlations between all evaluated parameters, regardless whether we evaluated the correlation of inflammatory infiltration with cytokine production, inflammatory infiltration with amastigote nests, or amastigote nests with cytokine production.

These data indicate a very important issue associated with *T. cruzi* infection in immunosuppressed individuals, including those undergoing cancer treatment, radiotherapy, and/or chemotherapy, as well as those affected by the presence of the HIV virus, wherein the immune deficiency is associated with persistent and worsening parasitic disease [46,47]; our data suggest an increased possibility of intestinal lesions in these individuals. Moreover, our data also contribute with recent findings showing that *Triatoma virus* is not an important factor to exacerbate Chagas disease when it’s transmitted by vectors since it does not replicate and contribute only to the parasite concentration during infection in the human host [48]. This information is very important because it robustly suggests that the number of parasites present at the time of infection is critical for triggering inflammatory processes in the intestine to the extent that this process will culminate in disease development and progression since host immune response is very important in the host-parasite relationship.

## Conclusions

This work demonstrates that Inoculum size correlates with parasitemia, Inflammation, number of amastigotes in the intestinal tissues and cytokine production in C57BL/6 mice infected with Y strain. These experimental data support the idea that the parasite load considerably influences the *T. cruzi*-induced intestinal inflammatory response and contributes to the development of the digestive form of the disease.

## Abbreviations

CD: Chagas disease
*T. cruzi*: *Trypanosoma cruzi*
HE: hematoxylin/eosin
min: minutes
DAB: diaminobenzidine
TNF-α: Tumor Necrosis Factors-alpha
IFN-γ: Interferon-gamma
IL-2: Interleukin-12
IL-6: Interleukin-6
IL-17: Interleukin-17
IL-10: Interleukin-10
